# A Treatise on the Malformation, Injuries, and Diseases of the Rectum and Anus

**Published:** 1837-10

**Authors:** 


### Art. XI.

*A Treatise on the Malformations, Injuries, and Diseases of the Rectum and Anus. Illustrated with Plates.* By George Bushe, m.d., formerly Professor of Anatomy and Physiology, &c.—*New York,* 1837. 8vo. pp. 299.

This treatise is a very respectable compilation, but not much more. It contains few original suggestions, and, mixed with a great deal of excellent but familiar advice, the reader will find some that is disputable. Some of the points which come under this last category we shall advert to in the present brief notice of its contents.

Dr. Bushe commences the practical part of his book with the subject of congenital imperfections. These he divides into obstructions, either at the anus or within the gut; deficiency, entire or incomplete, of the rectum; and false terminations of the rectum in other parts, and of other parts in the rectum.

Under the first head he mentions, in addition to commoner examples, an instance of *two congenital septa in the rectum.* This had fallen under his own observation. Dr. Bushe once saw a case of this kind in a new-born infant brought into the dissecting room. The upper part of the rectum was loaded with meconium; the partitions were thin and friable, being about three-quarters of an inch apart, while the lowermost was nearly half an inch from the anus.

Under the second head, he properly deprecates attempts to open the imperforate bowel from the loins or belly, when its termination cannot be found by dissection of the perineum, and search after its cul-de-sac in the pelvis.

Under the third head, which presents some perfectly remediable cases, our author underrates the extent of relief which the patient may obtain by proper management. The instances to which we refer are those in which the rectum, otherwise imperforate, terminates in the vagina. In these cases two things are to be done: one, to open a channel from the rectum into the perineum; the other, to close the communication with the vagina. Dr. Bushe, speaking of the first part of this process of restoration, observes, "After these operations, the new canal, being nothing more than a fistula, will always be liable to contract, and must, at best, perform its office very imperfectly." Perhaps many others are not aware how perfectly, on the contrary, the retentive and excretory functions may be performed under such circumstances. We have at present under our care a patient, twenty years of age, in whom the last two inches of the rectum are congenitally deficient; but, by constantly wearing a short metal bougie, this patient goes about with the same comfort, and is capable of the same bodily exertion and active habits as other people. No trouble arises from the want of a sphincter, although the diameter of the artificial channel is full half an inch: the bowels act readily and properly daily, after an injection of tepid water.

Foreign bodies obstructing the canal of the rectum, Dr. Bushe observes, are either feculent or alimentary concretions, or hard substances accidentally swallowed, or that have been introduced into the rectum; the latter are such as have either been passed into the bowel by the patient, to relieve obstinate costiveness, or (being knives, gallipots, portions of wood, and the like,) have been introduced "most commonly by wicked persons, who generally take advantage of the inebriated state of their intended victim." Dr. Bushe gives a good account of the various mechanical resources available in such cases; and the following example is a favorable instance of his illustrations of these diseases.

"The instruments necessary for extracting these bodies are, blunt hooks of different sizes and shapes, a lever, gimlet, cutting forceps, strong long scissors with probe points, a six inch narrow saw, wooden gergeret, polypus and lithotomy forceps of different shapes and sizes, a speculum, strong waxed ligatures, metallic tubes of various length and size, and a probe-pointed bistoury; to all of which, the crooked finger and a small hand are admirable adjuncts.

"When the foreign body is large or spiculated, it may be necessary to divide the sphincter, in order to seize and extract it safely. This, however, in consequence of its large size, can rarely be necessary, for the anus is very dilatable, as I had an opportunity of testing in the case of a delicate female, thirty-five years of age, who for seven years had been subject to constipation and repeated attacks of colic; the former had increased, attended with sickness of stomach, while the latter became more frequent, and from which she only experienced relief when her bowels were moved—a task not accomplished without the most painful efforts and very great difficulty, much cathartic medicine and powerful enemata being necessary in each succeeding attack. I was called to visit her in one of these paroxysms, and found her sallow, emaciated, and dejected. From the severe bearing-down pains, together with the sense of weight and fullness in the sacral region, which she complained of, I was led to make an examination of the rectum, when I found the mucous membrane slightly protruding from the anus, and very turgid, the sphincter excessively irritable, and a large concretion distending the pouch of the rectum. I now apprized her of the nature of the case, and the absolute necessity of removing the foreign body, to which she willingly consented. Having placed her hips over the edge of the bed, and bent her knees towards her chin, while she lay on her back, I introduced a strong and long lithotomy forceps, with which cautiously laying hold of the concretion, I slowly and steadily extracted it, with no more injury than slight laceration of the mucous membrane; although on measurement it proved to be six inches and three quarters in circumference, and two inches and a half in length. The bowels were then freely evacuated by injections; leeches and fermentations were applied to the anus, the recumbent position was enjoined, and a speedy recovery ensued." (P. 58.)

For the treatment of lacerations of the intestines in females, involving complete division of the sphincter, Dr. Bushe describes a new kind of pin for holding the torn edges in contact, which admits of being fixed by a screw. This instrument appears to us very well adapted for the object intended. Dr. Bushe has used it with success, both in recent and still granulating lacerations, and in those which have been some time cicatrized. In cases of the former class, he expresses a doubt of the necessity of the simpler operations, employed by Mr. Copeland and Mr. Mayo, of dividing the sphincter laterally, for the purpose of taking off the strain from the fissure; after which, in the experience of these surgeons, the rent spontaneously draws together, and unites by granulation; the only caution necessary being to keep the surfaces granulating for the three weeks which the laceration requires to heal. To us this operation appears likely to be more certain than any suture, however cleverly applied, to the edges of the recently torn part.

"The pin which I use is as thick as that used for hare-lip, and consists of three parts. The first, which is made of silver, is from one and a half to two inches long, curved as represented in the plate, terminating at one end in a female screw, and at the other in a transverse shoulder about a quarter of an inch long. The second is a triangular steel pin, exactly resembling that used for hare lip, and screws into the extremity of the first portion. The third is made of silver, and resembles the transverse shoulder of the first portion, with this exception, that a small male screw passes vertically from its centre, so that it may be fixed into the first portion, when the second is removed. This instrument is to be used in the following manner: the first and second portions being united, provided the tumefaction has nearly subsided, and granulations are formed, the patient should be brought to the edge of the bed, her hips elevated, and her knees approximated and carried towards the chin. The parts being now cleansed, the needle ought to be dipped in oil and inserted into the left side of the perineum, a line more than half the breadth of its curve from the edge of the wound, and immediately above the verge of the anus. When it has passed vertically for a distance equal to two-thirds of the depth of its curve, its point should be projected transversely, so as to cross the bottom of the wound, and then carried outwards through the other side of the perineum. This stage of the operation will be greatly facilitated: firstly, by pressing out the left labium during the transmission of the needle through the left portion of the perineum and the base of the wound; and secondly, by steadying the right side of the perineum, with the extremity of the thumb placed immediately without the point through which we desire the needle may pass. When the puncture has been completed, the steel pin should be unscrewed and the third portion fixed in its stead. If it be thought advisable to insert a smaller pin higher up, it may be done, and then a thread should be twisted over the extremities of one or both, as in the operation for hair-lip. It may be prudent to place a light bolster of lint beneath the twisted ligature." (P. 82.)

On the subjects of Fissures of the Rectum, Spasmodic Stricture of the Anus, and Ulcerations of the Rectum, Dr. Bushe does not add to our stock of information; and he makes some confusion by describing, under the first and second of these heads, the same disease. His account of Hemorrhoidal Tumours is good as far as it goes, but it does not contain a notice of all the varieties which this genus contains. He distinguishes, however, with judgment, hemorrhoidal congestion and hemorrhoidal bleeding from these tumours; and, with truth no doubt, observes that the varicose veins, which are often found in piles, and to which they have been attributed, are not an *essential* part of their structure.

"I have repeatedly injected these tumours with coloured water, both from the arteries and veins, and when cut into while the fluid was projected, small jets were observed to issue from many points. I have frequently dissected them with the greatest care, and found that they were spongy, reddish, and contained both arteries and veins, the latter being most capacious, but always perfectly healthy. Their surface is villous, and generally bleeds when touched roughly, or scratched with the nail, the blood which issues being of a florid red colour. In many instances, I have been able to rub off exceedingly vascular and fragile adventitious membranes from their superficies. Thus, it would seem, that they may acquire an increase of magnitude in this way." (P. 152.)

In treating of Prolapsus of the Rectum, Dr. Bushe falls into the common mistake of considering cases, in which the entire structure of the bowel is everted, as cases of protrusion of the mucous membrane only: his rules of treatment are the familiar ones. Upon Relaxation of the Anus, he is too brief: upon Relaxation of the Rectum, with partial contraction and invagination, he gives the following useful case.

"A few months ago, I attended a lady who laboured under this disease for six weeks before I was consulted. During this time she had colic pains, vomiting, constipation, and hysterical symptoms. She had repeated calls to stool, but very seldom discharged more than a sanguineous or puriform mucus, which, however, was rather abundant. She asserted that there was something within the gut, and attributed all her suffering to it. This led me to make an examination, when I found that the lower part of the pouch of the rectum was large and empty; but, by making her bear down, I perceived at once an invagination of the mucous membrane, which was rather firmer and harder than natural, with an opening in its centre, not much exceeding an inch in diameter. I ordered her a light diet, the horizontal position, a bluepill at night, and a teaspoonful of Epsom salt on the following morning. Provided her medicine did not operate by noon, an injection consisting of gruel and oil was administered. After her bowels were evacuated, I daily introduced into the rectum a gut nine inches long, and then inflated it: this she retained for an hour, when, the air being allowed to escape, it was withdrawn. Finally, alum dissolved in a decoction of galls was injected into the bowel every afternoon, and retained as long as possible. Under this treatment, she recovered in little more than a month." (P. 216.)

With the *malignant diseases* of the rectum Dr. Bushe appears to have been very imperfectly acquainted: he describes in a separate chapter Polypi of the Rectum, misled by the term to overlook the fact that they uniformly belong to the same family, and are a variety and a part of them. The account given of Stricture and Fistula is respectable, but no more; and the expanding bougie, which he recommends for the treatment of the former, is at once too complicated for general use, and mechanically too powerful to be employed with safety. He does not seem to be sufficiently aware that any forcible dilatation of stricture of the rectum is liable to bring on fatal peritonitis.

The plates which accompany this volume are extremely good, and calculated to convey to any one who has not seen the common varieties of hemorrhoidal disease a correct conception of them.

---

